# Analysis of the relationship between the *KRAS G12V* oncogene and the Hippo effector *YAP1* in embryonal rhabdomyosarcoma

**DOI:** 10.1038/s41598-018-33852-7

**Published:** 2018-10-23

**Authors:** Abdalla D. Mohamed, Nupur Shah, Simone Hettmer, Neil Vargesson, Henning Wackerhage

**Affiliations:** 1 University of Aberdeen, School of Medicine, Medical Sciences and Nutrition, Foresterhill, Aberdeen, AB25 2ZD Scotland; 2 Present Address: Technical University of Munich, Faculty of Sport and Health Sciences, Georg-Brauchle Ring 60-62, 80992 Munich, Germany; 3 Division of Pediatric Hematology and Oncology, Department of Pediatrics, University Medical Center Freiburg, Freiburg, Germany; 4 Present Address: Institute of Developmental Genetics Helmholtz Zentrum München, German Research Center for Environment and Health Ingolstaedter Landstrasse 1, D-85764 Munich, Neuherberg Germany

**Keywords:** Genetics research, Paediatric research

## Abstract

Persistent hyperactivity of the Hippo effector YAP in activated satellite cells is sufficient to cause embryonal rhabdomyosarcoma (ERMS) in mice. In humans, YAP is abundant and nuclear in the majority of ERMS cases, and high YAP expression is associated with poor survival. However, *YAP1* is rarely mutated in human ERMS. Instead, the most common mutations in ERMS are oncogenic RAS mutations. First, to compare *YAP1* *S127A* and *KRAS* *G12V*-driven rhabdomyosarcomas, we re-analysed gene expression microarray datasets from mouse rhabdomyosarcomas caused by these genes. This revealed that only 20% of the up or downregulated genes are identical, suggesting substantial differences in gene expression between YAP and KRAS-driven rhabdomyosarcomas. As oncogenic RAS has been linked to YAP in other types of cancer, we also tested whether *KRAS G12V* alone or in combination with loss of *p53* and *p16* activates YAP in myoblasts. We found that neither *KRAS G12V* alone nor *KRAS G12V* combined with loss of *p53* and *p16* activated Yap or Yap/Taz-Tead1–4 transcriptional activity in C2C12 myoblasts or U57810 cells. In conclusion, whilst oncogenic KRAS mutation might activate Yap in other cell types, we could find no evidence for this in myoblasts because the expression of *KRAS G12V* expression did not change Yap/Taz activity in myoblasts and there was a limited overlap in gene expression between *KRAS G12V* and *YAP1 S127A*-driven tumours.

## Introduction

Rhabdomyosarcoma is the most common soft tissue sarcoma in children. The two main subtypes are alveolar rhabdomyosarcoma (ARMS), which is associated with *PAX3/7-FOXO1* fusion genes, and embryonal rhabdomyosarcoma (ERMS), which is associated with mutations in common cancer genes such as *HRAS*, *KRAS*, *NRAS*, *CTNNB1*, *PIK3CA* and *TP53*^1^.

The ubiquitously expressed Hippo pathway is a set of proteins that have been discovered as a result of tumour suppressor screens in the fruitfly (*Drosophila melanogaster*). At the core of the mammalian Hippo pathway lie two kinases with two isoforms each: MST1 (gene symbol *STK4*), MST2 (gene symbol *STK3*), as well as LATS1 and LATS2. These kinases phosphorylate and inhibit the transcriptional co-factors YAP (gene symbol *YAP1*) and TAZ (gene symbol *WWTR1*). Active YAP and TAZ bind to TEAD1–4 transcription factors to regulate sets of genes that typically increase proliferation and organ size and that promote tumourigenesis^2^.

There is substantial but scattered evidence linking the Hippo pathway to sarcomas^3^. Transgenic mice with mutations of Hippo members (i.e. mutants of *Lats1*^4^, *Nf2/Merlin*^5^ and *Mob1*^6^) all spontaneously develop various types of sarcoma. We previously found that the expression of the constitutively active human mutant *YAP1* *S127A* in murine satellite cells causes ERMS-like tumours^7^. Additionally we found that YAP is nuclear and presumably active in 143 (≈73%) of 171 ERMS cases^7^. We also found that the abundance of the YAP paralogue TAZ in ERMS is associated with reduced survival and that TAZ can transform myoblasts^8^.

YAP and other Hippo members are rarely mutated in ERMS and other types of sarcoma^1,9^ as well as most other types of cancer^10^. If Hippo members are mutated, it is typically in the form of copy number aberrations. Specifically, copy number gains of *YAP1* and of *VGLL3* have been reported for sarcoma^7,11^. VGLLs and YAP can bind Tead transcription factors at the same site (demonstrated for Vgll1 and TEAD4^12^) suggesting that they are related transcriptional co-factors. However, the frequency of Hippo gene copy number aberrations and other mutations is too low to account for the common nuclear localization of YAP in human ERMS^7^, suggesting that mutated cancer genes cause the activation of YAP and TAZ.

Supporting this notion are studies that link oncogenic RAS isoforms^13–15^, WNT members^16^, and mTOR mutants^17,18^ to the Hippo pathway and especially YAP activity. For instance, YAP is essential for neoplastic initiation and progression of *KRAS*-mediated pancreatic ductal adenocarcinoma (PDAC)^15^. YAP also bypasses KRAS addiction in PDAC^13^, and functionally substitutes KRAS in murine lung cancer model^14^ and mediates drug resistance to drugs that target oncogenic RAS signalling in human non-small cell lung cancer cells^19^. In *KRAS G12V* expressing human myoblasts, YAP has been shown to promote cell proliferation and inhibit apoptosis and myogenic differentiation *in vitro* and in murine xenografts *in vivo*^20^.

Because recurrent mutations of some of these genes are frequently found in ERMS^1^, it seems likely that YAP is activated through cross-talk with frequently mutated cancer genes. In addition, expression of *KRAS G12V* and knockout of *p16* in isolated satellite cells results in the formation of rhabdomyosarcoma with pleomorphic features. The transcriptional profiles of these tumours recapitulate the gene expression signatures of human embryonal rhabdomyosarcoma^21^.

Based on the above, we hypothesize that oncogenic RAS mutations which are frequently found in ERMS^1^ activate YAP through unknown mechanisms. The aim of this project was therefore to test the hypothesis that *KRAS G12V* is sufficient to activate YAP in myoblasts and that YAP is essential for the tumourigenic effects of *KRAS G12V* in this cell type.

## Results

**Figure 1:**
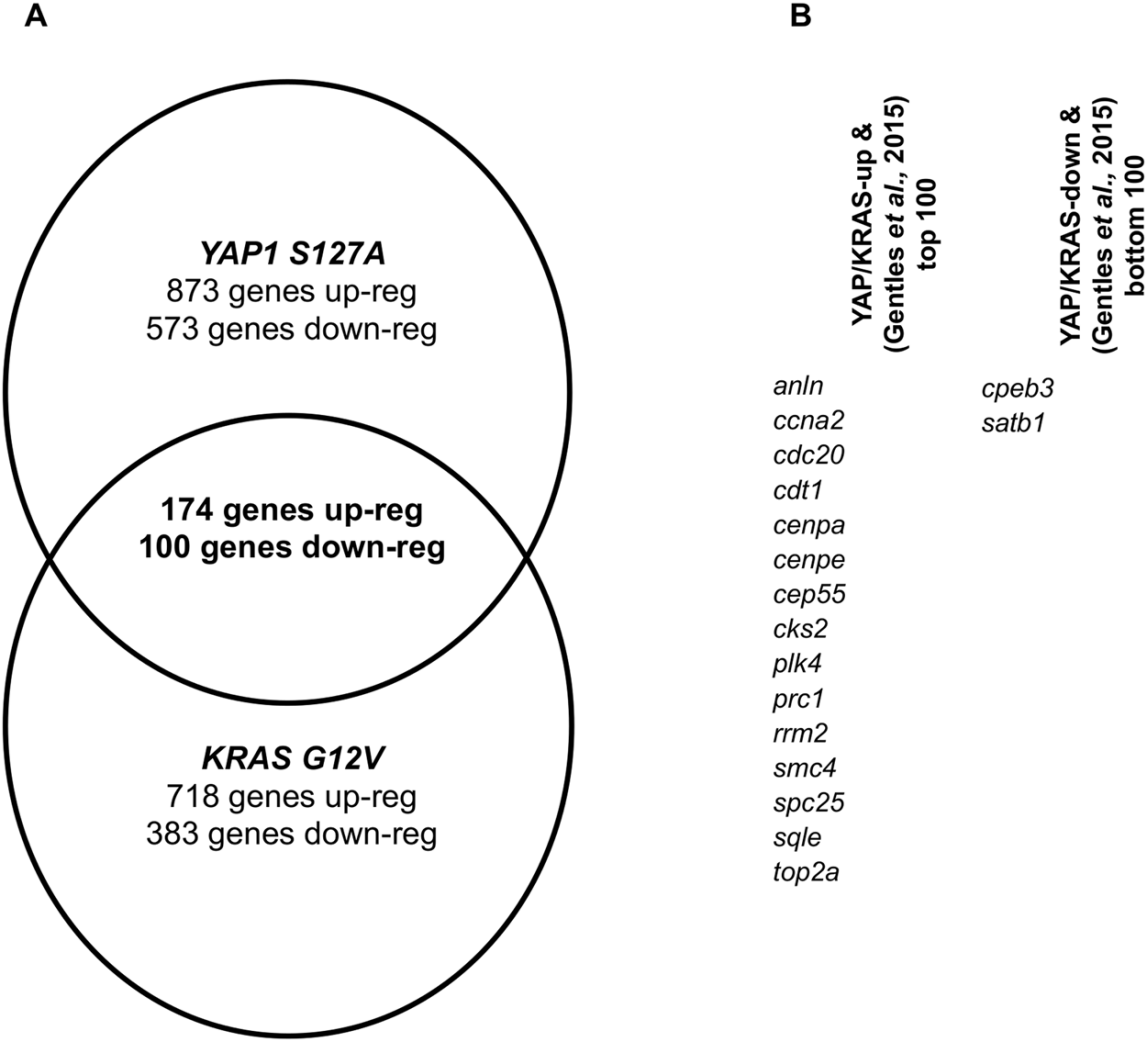
Gene overlap analysis of *YAP1* *S127A* and *KRAS* *G12V*-induced rhabdomyosarcomas. (**A**) Venn diagram displaying the number of genes modulated by *YAP1 S127A* and/or *KRAS G12V*- induced rhabdomyosarcomas versus differentiated skeletal muscle (Up-reg: up-regulated, down-reg: down-regulated). (**B**) Overlap of common up-regulated or down-regulated genes in both *YAP1 S127A* and *KRAS G12V*-induced rhabdomyosarcoma with top 100 or bottom 100 genes best associated with poor survival in 18,000 cases of human cancer.

The expression of *YAP1 S127A* alone in activated satellite cells results in the formation of ERMS in mice^7^. Also, *KRAS G12V* in combination with a *Cdkn2a* null mutation gives rise to pleomorphic rhabdomyosarcoma with a similar gene expression profile to embryonal rhabdomyosarcoma^21^ To investigate whether the gene expression in these two types of tumour is similar, we compared the gene expression profiles of the *YAP1 S127A*-driven ERMS^7^ with those of *KRAS G12V*-*Cdkn2a* null-driven ERMS^21^. This analysis revealed substantial differences in up-regulated genes between *YAP1 S127A* and *KRAS G12V*-induced rhabdomyosarcomas; approximately 20% (174 genes) of the genes, up-regulated in sarcomas compared to differentiated skeletal muscle, are identical in *YAP1 S127A*-driven and *KRAS G12V*-*Cdkn2a* null-driven ERMS (Fig. 1A).

To identify common features among the 174 genes that are up-regulated in both mouse RMS types we performed an enrichment analysis using TOPPgene (https://toppgene.cchmc.org/enrichment.jsp). This revealed that both *YAP1 S127A* and *KRAS G12V*-induced genes are associated with mitosis and the cytoskeleton. Genes induced in *YAP1 S127A* but not *KRAS G12V*-*Cdkn2a* null-driven ERMS are enriched for immune system-associated genes and the DNA packaging complex, as well as for nucleosomes and chromosomes. Genes induced by *KRAS G12V*-*Cdkn2a* null but not *YAP1 S127A*-driven ERMS are enriched for genes associated with cell differentiation, cell projections and neuronal differentiation plus junctions and focal adhesions (Table S4).

*YAP1 S127A* and *KRAS G12V*-*Cdkn2a* null-driven ERMS repress the expression of mostly different gene sets in sarcomas compared to differentiated skeletal muscle, but again approximately 20% (100 genes) of the down regulated genes are identical. An enrichment analysis of the 100 down-regulated genes revealed that *YAP1 S127A* and *KRAS G12V*-*Cdkn2a* null-repressed genes are enriched for differentiated skeletal muscle genes (Fig. 1A).

Next, we tested whether genes that are either up-regulated or repressed in both *YAP1 S127A* and *KRAS G12V*-*Cdkn2a* ERMS overlap with the top 100 genes, whose high or low expression has been associated with poor survival in 18,000 cases of human cancer^22^. 15 out of 174 genes (Fig. 1B) that are up-regulated in both *YAP1 S127A* and *KRAS G12V*-*Cdkn2a*-null induced rhabdomyosarcoma overlap with top 100 genes whose high expression is best associated with poor survival in 18,000 cases of human cancer. The enrichment is even greater in *YAP1 S127A*-driven ERMS, as 5 of *YAP1 S127A*-induced genes (*BIRC5, FOXM1, TOP2A, TPX2*, and *CCNB1*) are also genes whose high expression is most associated with poor survival across 18000 cases of human cancer^22^. These genes are associated with mitosis and cell proliferation. Only 2 (*CBEP3* and *SATB1*) out of 100 genes (Fig. 1B) that are down regulated in *YAP1 S127A* and *KRAS G12V*-*Cdkn2a*-null induced rhabdomyosarcoma overlap with the bottom 100 genes whose low expression is best associated with poor survival in 18,000 cases of human cancer.

We then asked if genes that are either up regulated or repressed in both *YAP1 S127A* and *KRAS G12V*-*Cdkn2a* ERMS overlap with the 260 genes that have been identified as “significantly mutated” in cancer using a MutSigCV analysis^10^. Only 2 (*CCNTB1* and *RUNX1*) out of 260 genes are genes that are both up-regulated by *YAP1 S127A* and *KRAS G12V*, and are significantly mutated in cancer^10^. In contrast, there is no overlap between genes that are repressed in both *YAP1 S127A* and *KRAS G12V*-*Cdkn2a* null-induced rhabdomyosarcoma, and genes that are significantly mutated in cancer^10^.

**Figure 2:**
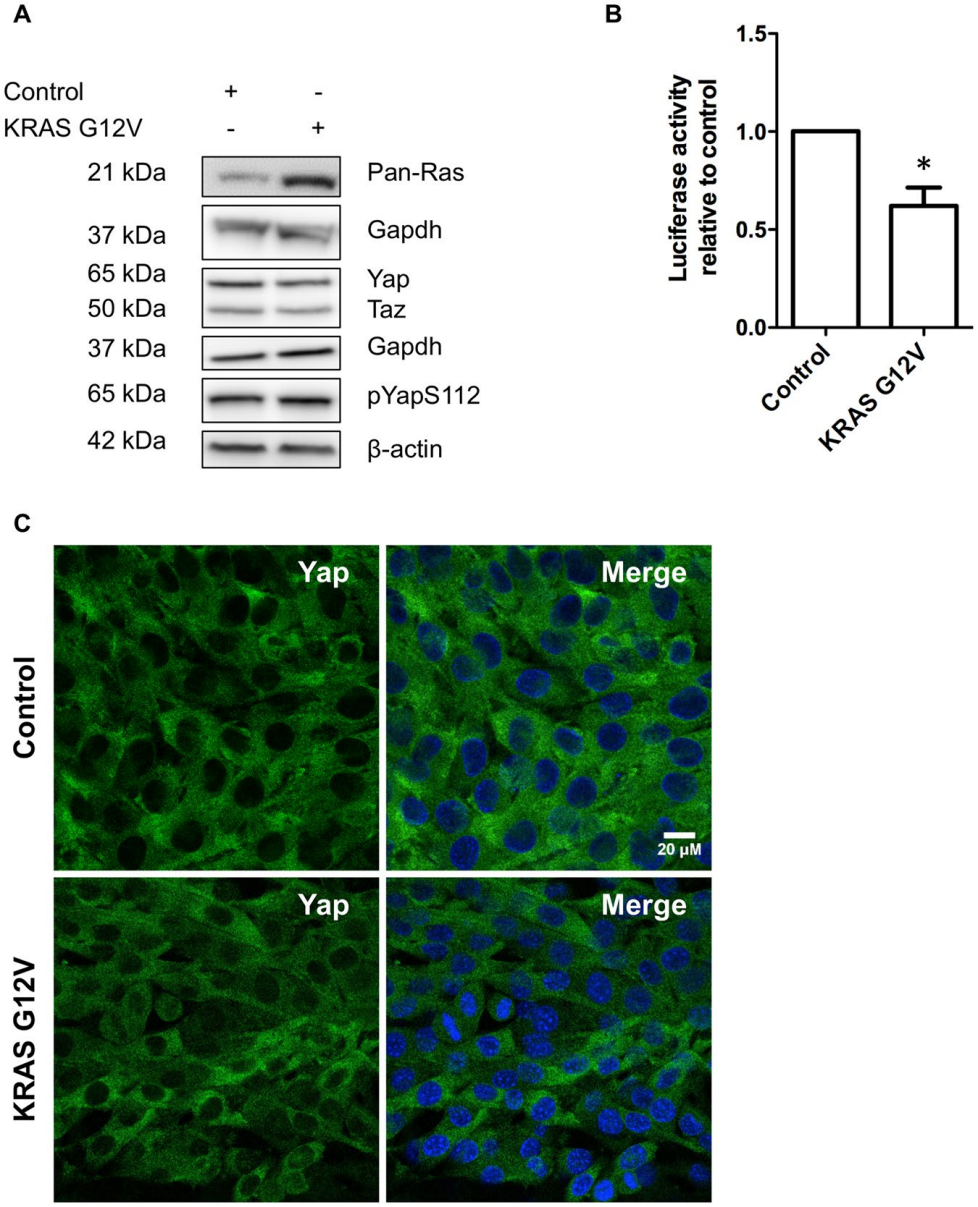
Expression of oncogenic *KRAS G12V* in C2C12 myoblasts does not change Yap/Taz activity. (**A**) Representative Western blots showing pan-RAS, total Yap, Taz, and phosphorylated Yap protein levels in C2C12 myoblasts, with relevant β-actin or Gapdh as loading controls. (**B**) Effect of *KRAS G12V* expression on 8xGTIIC Yap/Taz Tead1-4 luciferase reporter normalised to Tk-renilla in C2C12 myoblasts. Data is presented as fold change compared to control (mean ± SD) from 3 independent experiments where an asterisk denotes significant difference (p < 0.05) between control and *KRAS G12V* using a Student’s t-test. (**C**) Confocal images of C2C12 myoblasts immunolabelled with Yap (green) and DAPI (blue). Scale bar equals 20 μm.

Next, to investigate whether *KRAS G12V* can alter Yap/Taz abundance or activity-related phosphorylation, we transduced C2C12 myoblasts with retroviruses expressing either control vector or *KRAS G12V* and then analysed Yap/Taz through Western blotting and immunohistochemistry. As shown in (Fig. 2A), *KRAS G12V* expression did not change Yap/Taz protein levels nor Ser112 (paralogous to serine 127 in humans) phosphorylation of Yap. To test whether *KRAS G12V* affects Yap localisation, we seeded C2C12 myoblasts expressing *KRAS G12V* or empty vector at high confluence to promote cytosolic localisation^23^ to see whether *KRAS G12V* makes Yap translocate to the nucleus. We found that *KRAS G12V* did not promote the nuclear translocation of Yap (Fig. 2B) or Taz (Fig. S1).

Next, to test whether *KRAS G12V* expression affects Yap/Taz transcriptional activity, we transduced C2C12 myoblasts with control and *KRAS G12V* vectors and then used a 8xGTIIC Yap/Taz Tead1-4 reporter as a readout for the transcriptional activity^24,25^. Measuring normalised luciferase activity from the Hippo reporter construct revealed that *KRAS G12V* expression significantly reduced Yap/Taz Tead1-4 transcriptional activity (Fig. 2C). Collectively, these data suggest that *KRAS G12V* alone does not activate Yap or Yap/Taz-Tead1-4 transcriptional activity in C2C12 myoblasts.

**Figure 3:**
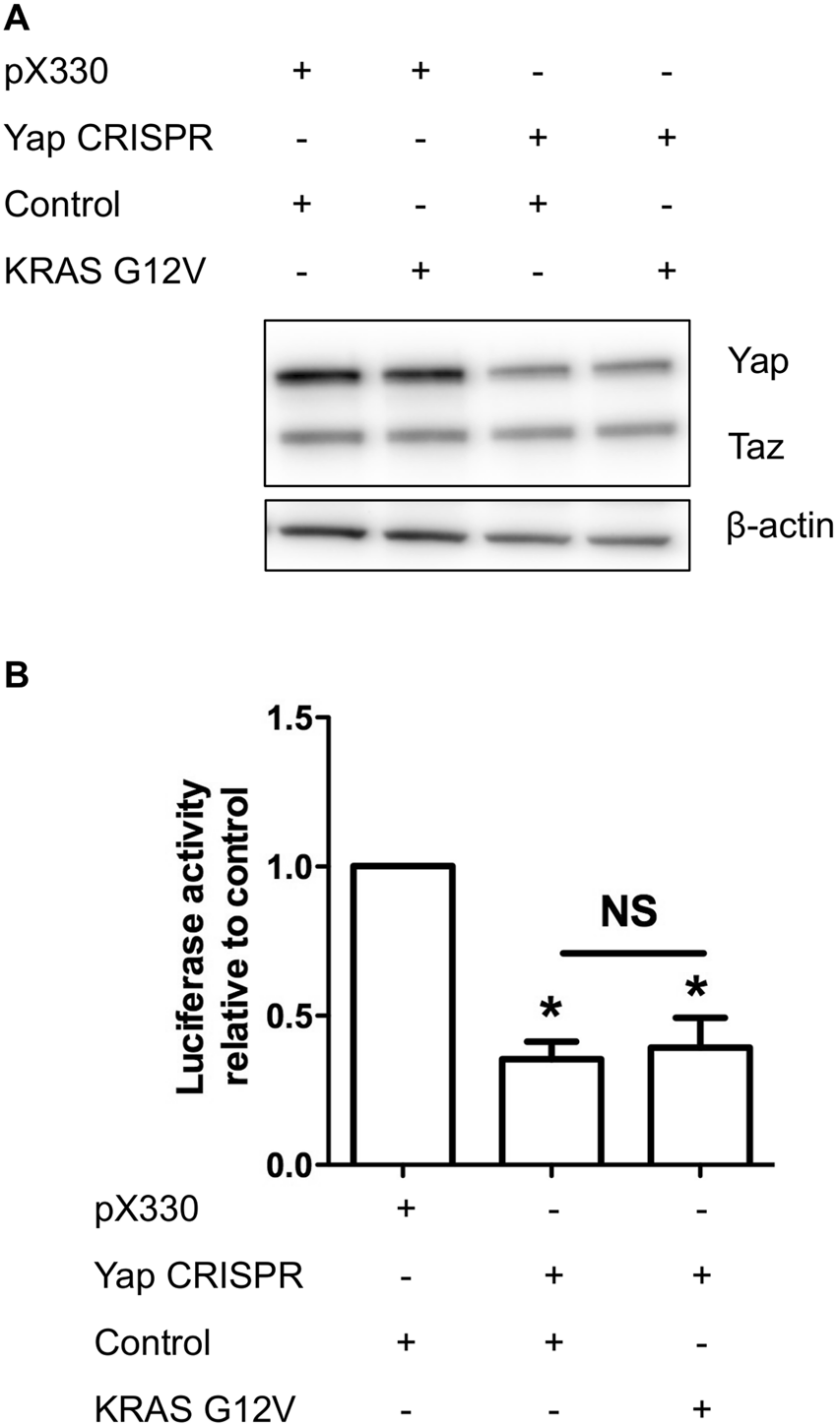
Expression of *KRAS G12V* does not compensate for Yap knockdown in C2C12 myoblasts. (**A**) Representative Western blots showing total Yap and Taz protein levels in C2C12 myoblasts, with β-actin as a loading control. (**B**) Effect of *KRAS G12V* expression on 8xGTIIC Yap/Taz Tead1-4 luciferase reporter in C2C12 myoblasts with low Yap activity (n = 3). Data is represented as (mean ± SD) from 3 independent experiments where an asterisk denotes significant difference (p < 0.05) between control, *KRAS G12V* or control and Yap knockdown and NS (non-significant) between *KRAS G12V* and *Yap* knockdown using a Student’s t-test.

We also considered that the effects of *KRAS G12V* expression on Yap protein levels might be masked by high Yap activity because of the high serum content of the culture media^26^. We therefore knocked down Yap, which reduced Yap protein level (Fig. 3A) as well as Hippo reporter activity (Fig. 3B), and then transduced the cells with control vector or *KRAS G12V* retroviruses, However*, KRAS G12V* expression did not compensate the reduction in Yap protein levels and the Hippo reporter activity (Fig. 3A,B).

**Figure 4:**
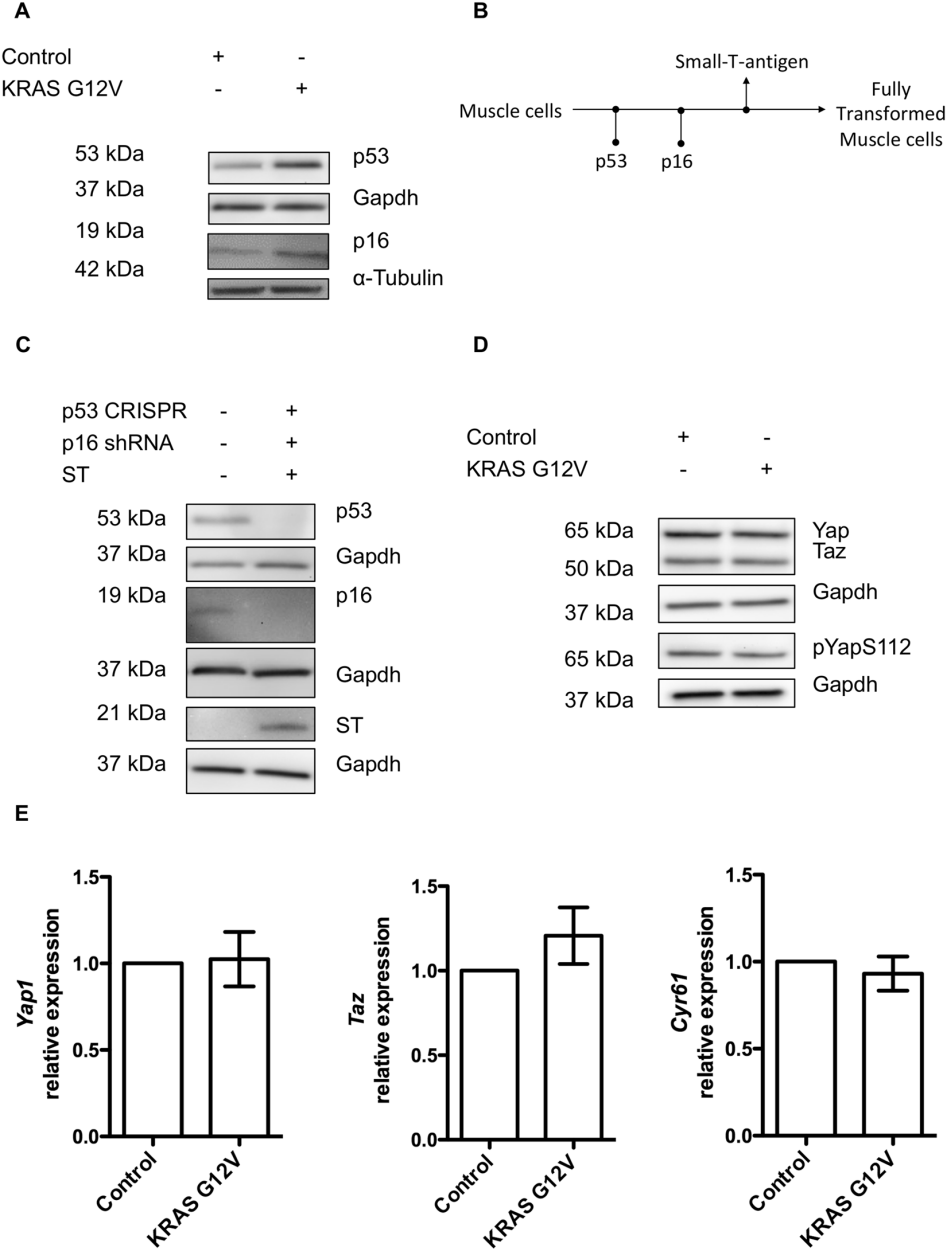
Expression of *KRAS G12V* does not change Yap/Taz activity in genetically manipulated C2C12 myoblasts (p53 and p16 knockdown and small-T-antigen overexpression). (**A**) Representative Western blots showing p53 and p16 protein levels in C2C12 myoblasts after the expression of *KRAS G12V*, with relevant α-Tubulin or Gapdh as loading control (p16 western blot is imaged at high exposure). (**B**) Diagram of genetic manipulations performed to establish stable C2C12 myoblasts cell line with loss of p53 and p16 and small-T-antigen overexpression. (**C**) Representative Western blots to validate p53 and p16 knockdown and small-T-antigen overexpression in C2C12 myoblasts, with Gapdh as loading control (p16 western blot is imaged at high exposure). (**D**) Effect of *KRAS G12V* expression on Yap, Taz and phosphorylated Yap S112 protein levels in genetically manipulated C2C12 myoblasts. (**E**) *Yap*, *Taz* and *Cyr61* mRNA expression after *KRAS G12V* expression. Expression is fold change compared to control vector (n = 3). Data is represented as (mean ± SD) from 3 independent experiments.

Stable expression of *HRAS G12V* induces cell senescence in primary human skeletal muscle cells^20,27^. To test whether *KRAS G12V* activates senescence signalling, we expressed either empty vector or *KRAS G12V* in C2C12 myoblasts and then measured p53 and p16 as senescence-associated proteins. We found that *KRAS G12V* significantly increased both p16 and p53 protein levels in C2C12 myoblasts^28^ when compared to the empty vector control (Fig. 4A). This suggests that *KRAS G12V* induces senescence-associated signalling in C2C12 myoblasts and this signalling might prevent the activation of Yap.

Therefore, to test the combinatorial effects of *KRAS G12V* expression and loss of senescence checkpoints, we knocked down p53 and p16 using CRISPR-cas9 and shRNA, respectively. We then overexpressed the small-T-antigen to generate a stable cell line with these mutations and verified the experimental manipulations by western blotting (Fig. 4B). We then expressed either control vector or *KRAS G12V* or empty vector in the genetically modified C2C12 myoblasts. As shown in (Fig. 4D), *KRAS G12V* expression did still not change Yap/Taz protein levels or the phosphorylation of Yap. Additionally, there was no change in the mRNA levels of *Yap1*, *Wwtr1* (encoding Taz) or the Hippo target gene *Cyr61* as shown by qPCR (Fig. 4E). Together, this suggests that even the expression of *KRAS G12V* together with the suppression of senescence checkpoints and small-T-antigen expression does not suffice to increase Yap/Taz Tead1-4 activity in myoblasts as judged by several assays.

**Figure 5:**
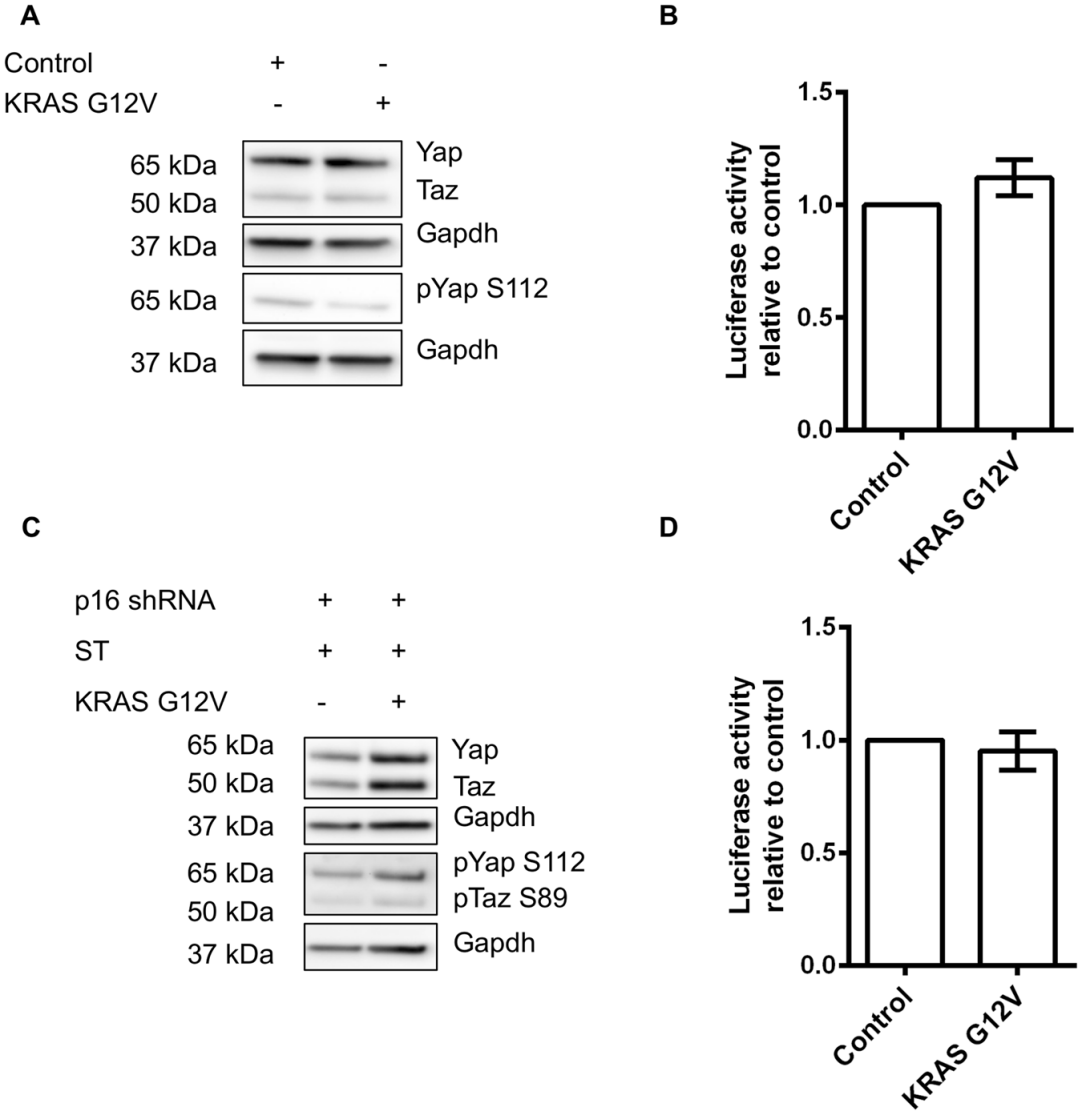
Expression of *KRAS G12V* alone or in combination with *p16* loss and small-T-antigen expression in U57810 cells does not change Yap/Taz transcriptional activity. (**A**) Representative Western blots showing total Yap, Taz, and phosphorylated Yap levels in U57810 cells, with Gapdh as loading control. (**B**) Effect of *KRAS G12V* expression on 8xGTIIC Yap/Taz Tead1-4 luciferase reporter normalised to Tk-renilla in U57810 cells. Data is presented as fold change compared to control (mean ± SD) from independent experiments. (**C**) Representative Western blots showing total Yap, Taz, and phosphorylated Yap S112/Taz S89 levels in U57810 cells, with Gapdh as a loading control. (**D**) Effect of *KRAS G12V* expression combined with p16 loss and small-T-antigen expression on 8xGTIIC Yap/Taz Tead1-4 luciferase reporter normalised to Tk-renilla in U57810 cells. Data is presented as fold change compared to control (mean ± SD) from 3 independent experiments.

To test whether this effect is specific to C2C12 myoblasts, we used U57810 cells, which were isolated from ERMS tumours arising in transgenic *Myf6*-Cre*/p53*^−/−^ mice^29^. We first transduced these cells with control vector and *KRAS G12V* retroviruses. As shown in (Fig. 5A), there was no change in Yap, Taz or Yap S112 phosphorylation protein levels or Hippo reporter activity (Fig. 5B). We then generated a stable cell line as described previously (Fig. 4A) by knocking down p16 and overexpressed small-T-antigen before transducing the cells with either control vector or *KRAS G12V* retroviruses. Here, *KRAS G12V* significantly increased Yap/Taz protein levels when compared to control. However, there was a proportional increase in the phosphorylation of Yap at the inhibitory S112 site (Fig. 5C). Furthermore, there was no change in the Hippo reporter activity in *KRAS G12V*-transduced cells (Fig. 5D), suggesting that Yap/Taz-Tead1-4 activity was not increased. In summary, none of the experimental variations of *KRAS G12V*, small-T-antigen*, p53* and *p16* was sufficient to increase Yap/Taz- TEAD1-4 activity in C2C12 and U57810 myoblasts.

## Discussion

A key dilemma in embryonal rhabdomyosarcoma is that YAP is expressed, nuclear and presumably active in the majority of human ERMS cases^7^ but that *YAP1* is not significantly mutated in human ERMS which has a high frequency of oncogenic RAS mutations^1^ Given that interactions between mutated, oncogenic RAS and YAP have been reported for other cell and cancer types^13–15^, we tested for evidence that mutated, oncogenic *KRAS* can activate YAP in (rhabdo-) myoblasts to regulate the expression of a shared set of genes. Our main finding is that *YAP1 S127A* and *KRAS G12V*-*Cdkn2a*-driven rhabdomyosarcomas only share 20% of the up or down-regulated genes and that the expression of *KRAS G12V* together with various genetic manipulations of myoblasts does not activate Yap in these cells. This differs substantially from other types of cells and cancer, where oncogenic RAS isoforms cross-talk to and activate YAP^13–15^.

Reanalysis of gene datasets of *YAP1 S127A*-driven ERMS and *KRAS G12V*-*Cdkn2a* null-driven ERMS in mice showed that only 20% of up and down-regulated genes are the same in *YAP1 S127A*-driven ERMS and *KRAS G12V*-*Cdkn2a* null-driven ERMS^7,21^. The *YAP1* *S127A* and *KRAS* *G12V* up-regulated genes include many genes involved with mitosis and the cell cycle such as the topoisomerase-encoding gene *Top2a*, centromere-related genes (*Cenpa, Cenpe*), or *Plk4* which regulates centriole biogenesis^30^. Of these genes, *Top2a* was independently identified in a ChIP-Seq analysis as a direct YAP/TAZ target^31^. Additionally, *YAP1 S127 and KRAS G12V* up-regulated genes are enriched for genes that encode members of the cytoskeleton or focal adhesions such as the filamin *FlnB*, the actinin *Actn1* or the actin-regulatory gene *Capg*. Conversely, *YAP1 S127 and KRAS G12V* down-regulated genes are often genes that are expressed in differentiated muscle such as the tropomyosin *Tpm3*, the myosin leight chain *Mylpf* and myoplalladin (*Mypn*). Collectively, this suggests that YAP and KRAS together both regulate the rhabdomyosarcoma core gene expression programme, which are genes associated with sustained proliferation whilst blocking terminal differentiation.

The role of the YAP and KRAS up-regulated cytoskeleton and focal adhesion-associated genes is less clear. However, there are some links between the cytoskeleton genes and Yap as the extra cellular matrix stiffness regulate Yap/Taz activity through cytoskeleton genes such as Rho and Actin^25^ and as Yap activity functions as mechano-regulator and influences the tissue tension^32^.

We additionally overlapped the *YAP1 S127 and KRAS G12V* up or down-regulated genes with the top or bottom 100 genes whose high or low expression is associated with poor survival in 18,000 cases of human cancer, respectively^22^. This is presumably the largest dataset linking the expression of individual genes to survival data pan-cancer. The overlap was 15 of the up-regulated and 2 of the down-regulated genes, suggesting that *YAP1 S127 and KRAS G12V* up-regulated mitosis and cell cycle-associated genes whose high expression is associated with poor survival pan-cancer. There was minimal overlap between *YAP1 S127 and KRAS G12V*-genes and genes that are significantly mutated pan cancer^10^.

Collectively, these findings reveal some limited similarities between *YAP1 S127A* and *KRAS G12V*-regulated genes but also that ≈80% of the target genes do not overlap. This contrasts other types of cancer where KRAS and YAP share a high proportion of target genes and are interchangeable. For example, in HCTtetK pancreatic adenocarcinoma cells, the expression of a mutant form of *YAP1* rescues the expression of 77% of the genes that were decreased following *KRAS* knockdown^14^ suggesting that *YAP* and *KRAS* mostly regulate the expression of the same set of genes (77%) in pancreatic ductal adenocarcinoma (PDAC).

In contradiction to recent studies that linked Ras signalling and the Hippo pathway in a subset of tumours and cell lines^33,34^, the expression of *KRAS G12V* alone or in combination with loss of *p53* and *p16* was not sufficient to activate Yap in C2C12 myoblasts or in U57810 RMS cells. YAP is induced in a subset of *KRAS*-driven PDAC tumours following knockdown of *KRAS* and supports the growth of these tumours^13^. Additionally, in *KRAS*-driven lung cancer, acquired resistance to KRAS suppression was associated with increased YAP activity^14^.

Here, we found that ectopic expression of *KRAS G12V* reduced the transcriptional activity of Yap in C2C12 myoblasts as measured by Yap/Taz Tead1-4 luciferase reporter by 25% compared to control cells suggesting that *KRAS G12V* may negatively regulate Yap activity. However, we did not observe any changes in total Yap/Taz protein levels or their phosphorylation. In addition, there was no change in the localisation of Yap/Taz in C2C12 myoblasts cultured at high density. We hypothesized that the reduction in Yap/Taz transcriptional activity may be because the expression of RAS in human muscle cells has been shown to activate senescence check points as judged by increased p53 and p16 protein levels^27,28^. We indeed observed that the expression of both proteins (p53 and p16) is increased following the ectopic expression of *KRAS G12V*.

We therefore assumed that it is essential to knockdown both p53 and p16 in order to obtain a fully transformed cell line. We based that assumption on previously published studies where (H,K,N) RAS oncogenes in the presence of small-T-antigen overexpression and knockdown p53 and p16 knockdown have been shown to control YAP protein turnover and stability independently of the Hippo pathway in human primary BJ fibroblasts and human mammary epithelial cells^33,34^. The combination of both *KRAS G12V* and small-T-antigen expression in addition to *p53* loss and *p16* loss did not change Yap/Taz activity in C2C12 myoblasts. We speculate that Yap may not be directly activated by oncogenic *KRAS* but that Yap still is required for *KRAS*-mediated tumourigenesis. This is because Yap protein levels in AT1 cells isolated from *KRAS*-driven lung cancer were indistinguishable from control cells after *KRAS* induction for 6 weeks. However, at later stages Yap levels varied and correlated with tumour cell proliferation^35^.

In U57810 cells, which are murine ERMS cells isolated from transgenic *p53* null mice in Myf6-positive differentiating myoblasts, the expression of *KRAS G12V* and small-T-antigen in addition to *p16* knockdown led to an increase in Yap/Taz protein levels but there was a proportional increase in Yap S112/Taz S89 phosphorylation. In addition, there was no change in Yap/Taz transcriptional activity measured by Hippo Yap/Taz Tead1-4 luciferase reporter or Yap/Taz mRNA levels. This suggests that Yap/Taz are still under the control of upstream regulators of the Hippo pathway and that additional genetic alterations may be required in order to establish a direct link between Ras and Hippo signalling pathways. This also highlights the cell-type dependent regulation of the Hippo pathway^36^.

In summary, whilst there is evidence for a direct relationship between KRAS mutations and YAP activation in colon, pancreatic and lung cancer, this is not the case in (rhabdo-myoblasts) as *KRAS G12V* and *YAP1 S127A* drive mostly different gene expression programmes and that *KRAS G12V* expression alone or in combination of other genetic variations does not activate Yap/Taz in myoblasts.

## Methods

### Gene overlap analysis

The method of overlap analysis and of other bioinformatical analyses is described in the supplementary methods.

### Cell culture

C2C12, mouse ERMS U5781 (isolated from transgenic mice harbouring loss of *p53* in Myf6-positive differentiating myoblasts^29^) and HEK293T cells were cultured in Dulbecco’s minimum essential medium (DMEM, Sigma) supplemented with 10% foetal calf serum (Hyclone) and passaged when needed.

### Retroviral and lentiviral expression vectors and transduction methods

pBABE puro *KRAS G12V* (Addgene plasmid #9052) and pBabe GFP Small-T-antigen (Addgene plasmid #10673) were used for the expression of *KRAS G12V* and Small-T-antigen respectively. pBABE-puro (Addgene plasmid #1764) was used as a control vector. *p16* shRNA oligo (TRCN0000231227, Table S1 in supplementary methods) was designed using the Broad Institute RNAi consortium (http://portals.broadinstitute.org/gpp/public/seq/search), was used for *p16* knockdown and subcloned into pLKO.1-blast, which was a gift from Keith Mostov (Addgene plasmid #26655). Retroviruses and lentiviruses were packaged in HEK293T cells as described previously^37,38^. Cells were transduced by incubation in diluted viral supernatant (1:4) until assayed.

### Crispr-Cas9 knockdown of p53 and Yap

Protospacer sequences of CRISPR/Cas9 against *p53* and *Yap* were designed by CRISPR DESIGN (http://crispr.mit.edu/). All specific target sequences were cloned into pX459 or pX330 vectors (Addgene plasmid #62988 and #42230 respectively). and verified by DNA sequencing. After the transient transfection of CRISPR/Cas9 into cells using Lipofectamine LTX reagent (Life Technologies), transfected cells were selected by DMEM culture medium supplemented with puromycin (2 μg/mL) (Sigma) for 48 hours and then expanded in regular culture medium. sgRNA oligo sequences targeting *p53* and *Yap* are listed in (Table S2, supplementary methods).

### Western blotting

Cells were lysed in modified RIPA buffer supplemented with protease and phosphatase cocktail inhibitors (Sigma). Whole cell lysate were separated via SDS-PAGE electrophoresis and transferred to 0.2 μM PVDF Western blotting membrane. Membranes were then probed with mouse anti-Yap/Taz (Santa Cruz, 101199, 1:100), rabbit anti-phospho Yap Ser127 (Cell Signaling, #9411, 1:1000) (this antibody can detect murine pYap S112 and pTaz S89^25^), pan-Ras (BD Bioscience, #610001, 1:1000), p53 (Cell Signaling, #2524, 1:1000), p16-INK4A (Proteintech, #10883-1-AP, 1:500), small-T-antigen (Millipore, #D01, 1:1000), tubulin (Sigma, #B512, 1:2000), actin (Sigma, #A5060, 1:2000) and Gapdh (Abcam, #Ab8245, 1:10000). Primary antibody binding was then visualised using species-specific conjugated secondary antibodies (Invitrogen) and digital imaging.

### Immunocytochemistry

Fixed cells were permeabilized with 0.5% (v/v) Tween-20 in PBS for 6 minutes and blocked with 20% (v/v) goat serum in PBS for 30 minutes. Cells were incubated with primary anti-Yap (Santa Cruz, #101199, 1:50) and anti-Taz (Sigma, #HPA007415, 1:50) antibodies overnight at 4 °C. Species-specific fluorochrome conjugated secondary antibodies (Alexafluor, Invitrogen, 1:500) were added for 90 minutes at room temperature, and the slides were mounted with Vectashield medium with DAPI (Vector laboratories).

### RNA extraction and quantitative RT-PCR

Total RNA was isolated from cultured cells using Trizol reagent (Invitrogen). cDNA was synthesized by reverse transcription using Superscript II (Invitrogen) and subjected to real-time PCR with gene-specific primers in the presence of 1x Light Cycler 480® SYBR green master (Roche). Relative abundance of mRNA was calculated by normalization to *Gapdh* (Table S3, supplementary methods).

### Luciferase reporter assays

For the luciferase reporter assay, retrovirally transduced C2C12 myoblasts were seeded in 6-well plates overnight. The 8xGTIIC Yap/Taz Tead1-4 luciferase reporter (Addgene plasmid #34615) was co-transfected with TK-renilla plasmid. 24 hours after transfection, we lysed the cells and measured luciferase activity using a Dual-Luciferase Reporter Assay System (Promega) following the manufacturer’s instructions. All luciferase activities were normalized against renilla activity.

### Statistical analysis

We analysed the data through GraphPad Prism (v5.0, GraphPad Software) and present the data as means ± S.D. Statistical comparisons were done using an unpaired Student’s-t-test. For all experiments, statistical analysis was conducted on raw data collected from at least three independent experiments performed on different occasions with three replicates each. A p-value of 0.05 or less was considered significant.

## Electronic supplementary material

Supplementary information

Full western blots

Supplementary dataset
