# Supplementary material for: Analysis of the relationship between the KRAS G12V oncogene and the Hippo effector YAP1 in embryonal rhabdomyosarcoma
Source: Sci Rep. 2018 Oct 23;8:15674. doi: 10.1038/s41598-018-33852-7 (PMC6199242; doi:10.1038/s41598-018-33852-7)
Supplement: Supplementary file 1 — Supplementary information [file 41598_2018_33852_MOESM1_ESM.pdf]

Analysis of the relationship between the *KRAS G12V* oncogene and the Hippo effector *YAP1* in embryonal rhabdomyosarcoma

Abdalla Mohamed, Nupur Shah, Simone Hettmer, Neil Vargesson, Henning Wackerhage

Supplementary information

Gene overlap analysis

We retrieved data from four publications and Gene Omnibus as detailed below:

We downloaded gene expression omnibus dataset GSE55186 and used genes whose expression was >5-fold up (Yap-up) or down (Yap-down) in YAP1 S127A-driven embryonal rhabdomyosarcoma when compared to skeletal muscle, respectively [1]. The fold-changes were subjectively chosen to obtain similarly large gene expression lists.

We downloaded gene expression omnibus dataset GSE22841. We used genes whose expression was either >12-fold up (Kras-up) or down (Kras-down) when compared to skeletal muscle, respectively [2].

We have downloaded supplementary data 1 (an Excel file) which contains PRECOG meta-z matrix values [3]. These values indicate the association between the expression of a gene and survival for 18,000 cases of human cancer. From this file we have used the top (i.e. whose high expression is most associated with poor survival pan-cancer) and bottom 100 genes (i.e. whose high expression is most associated with good survival pan-cancer), respectively.

We have used supplementary table 2 which contains the 260 significantly mutated cancer genes identified by analysis with the MutSig suite [4].

Data analyses:

We performed the following overlap using http://jura.wi.mit.edu/bioc/tools/compare.php:

a) Overlap of Yap-up (872 genes) and Kras-up (717 genes)
b) Overlap of Yap-down (411 genes) and Kras-down (313 genes)
c) Overlap of Yap/Kras-up & Gentles top 100 genes
d) Overlap of YapKras-down & Gentles bottom 100 genes
e) Overlap of Yap/Kras-up & Lawrence "significantly mutated in cancer" genes (260 genes)
f) Overlap of Yap/Kras-down & Lawrence "significnatly mutated in cancer" genes (260 genes)

With Yap/Kras-up and Yap/Kras-down gene lists we additionally performed a string analysis (https://string-db.org/) to illustrate functional interactions between the proteins.

To identify common functions of Yap and Kras induced or repressed genes we additionally performed a Toppgene functional enrichment analysis (https://toppgene.cchmc.org/).

Table S1. Catalogue number and the target shRNA sequences of scramble control shRNA and TAZ shRNA. All plasmids were obtained from addgene.

Catalogue No. / Clone No.	Target shRNA sequences
SHC002 (Scramble shRNA)	CCGGCAACAAGATGAAGAGCACCAACTCGAGTTGGTGCTCTTCATCTTGTTGTTTTT
TRCN0000370007 (p16 shRNA)	CCGGTCAAGACATCGTGCGATATTTCTCGAGAAATATCGCACGATGTCTTGATTTTTG

Table S2. sgRNA target site sequences.

Target gene	20nt sequence (5’ to 3’)	PAM	Strand
p53	CCTCGAGCTCCCTCTGAGCC	AGG	+
Yap	ACCAGGTCGTGCACGTCCGC	GGG	+

Table S3. Primers used for qPCR

Gene name	Primer	Primer sequence (5’ to 3’)
Yap	Forward	AAATGCTCCAAAATGTCAGGA
Yap	Reverse	CATTCGGAGTCCCTCCATC
Taz	Forward	TGCTACAGTGTCCCCACAAC
Taz	Reverse	TGACCGGAATTTTCACCTGT
Cyr61	Forward	GGATCTGTGAAGTGCGTCCT
Cyr61	Reverse	CTGCATTTCTTGCCCTTTTT

References

1. Tremblay, A. M. *et al.* The Hippo transducer YAP1 transforms activated satellite cells and is a potent effector of embryonal rhabdomyosarcoma formation. *Cancer Cell* **26,** 273–287 (2014).
2. Hettmer, S. *et al.* Sarcomas induced in discrete subsets of prospectively isolated skeletal muscle cells. *Proc. Natl. Acad. Sci.* **108,** 20002–20007 (2011).
3. Gentles, A. J. *et al.* The prognostic landscape of genes and infiltrating immune cells across human cancers. *Nat. Med.* **21,** 938–945 (2015).
4. Lawrence, M. S. *et al.* Discovery and saturation analysis of cancer genes across 21 tumour types. *Nature* **505,** 495–501 (2014).

Fig.S1 Confocal images of C2C12 myoblasts immunolabelled with Yap (green), Taz (red) and DAPI (blue). Scale bar equals 20 μm.

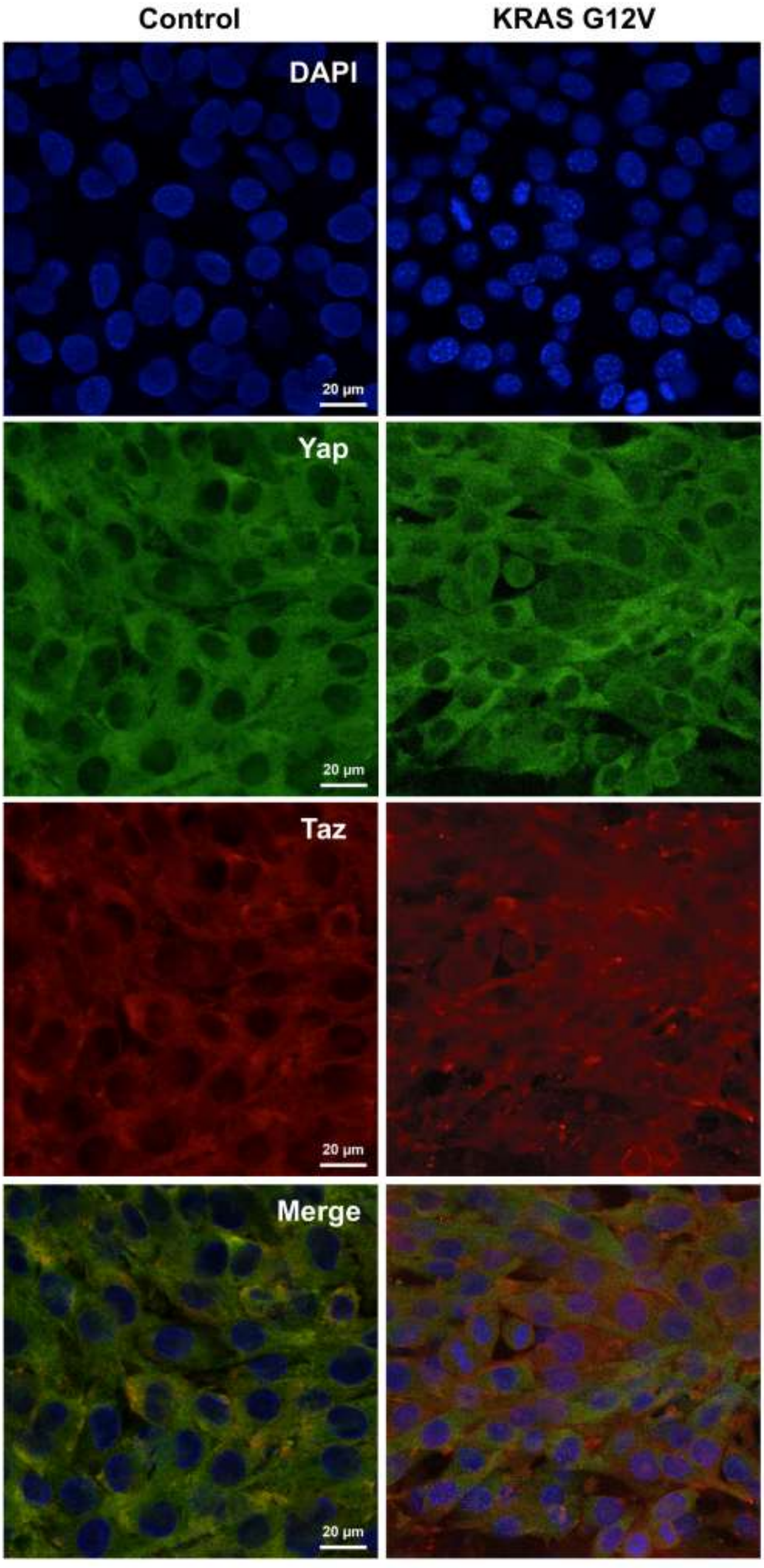

Control
KRAS G12V
DAPI
Yap
Taz
Merge
20 µm
20 µm
20 µm
20 µm
