# Supplementary figures and images for: Analysis of the relationship between the KRAS G12V oncogene and the Hippo effector YAP1 in embryonal rhabdomyosarcoma
Source: Sci Rep. 2018 Oct 23;8:15674. doi: 10.1038/s41598-018-33852-7 (PMC6199242; doi:10.1038/s41598-018-33852-7)

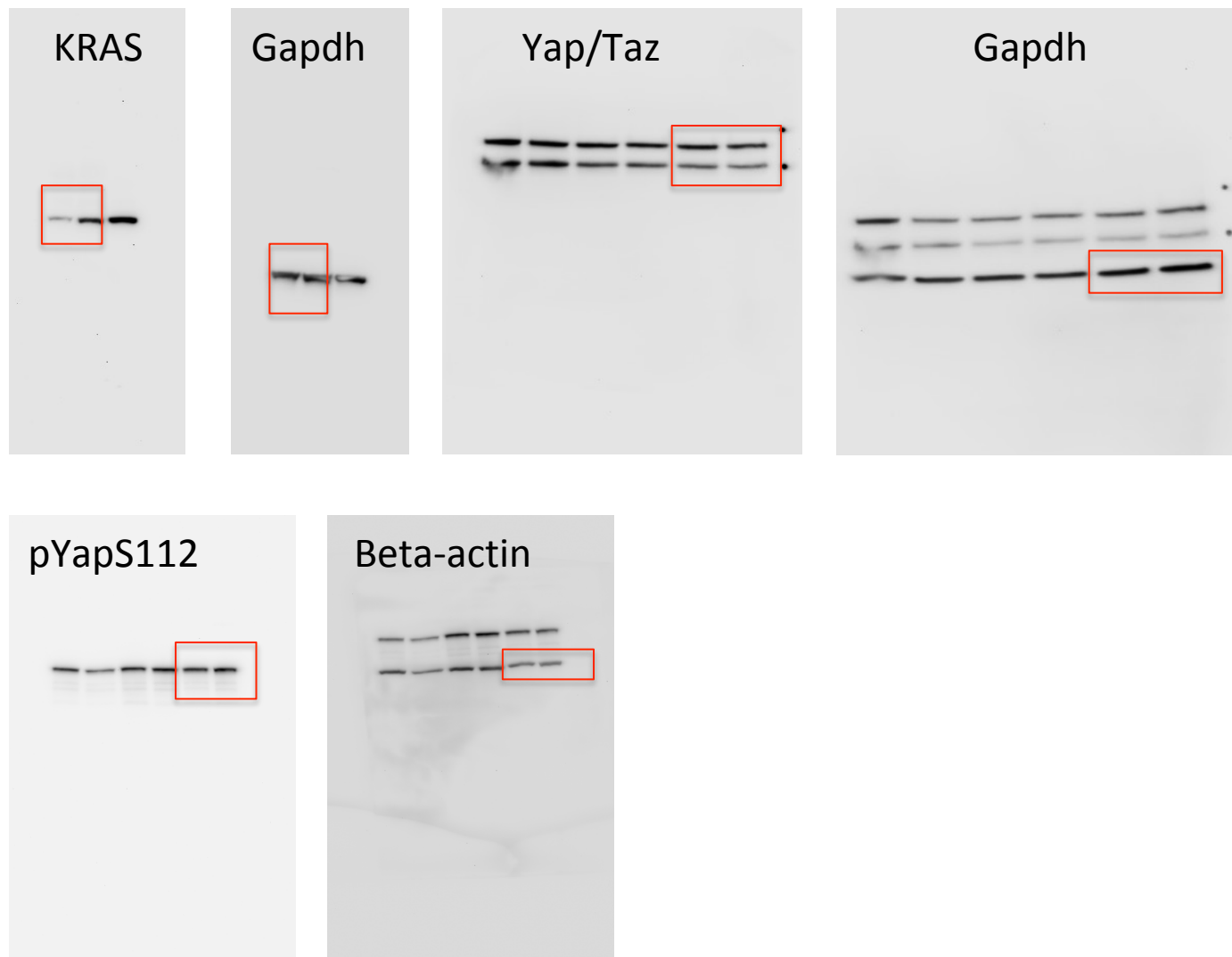

Figure.1A

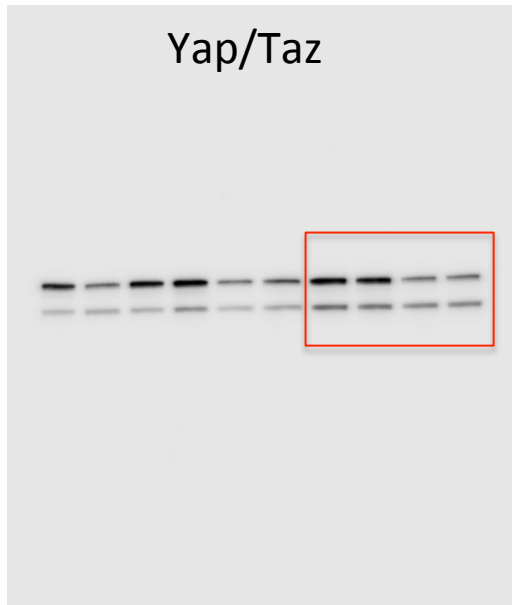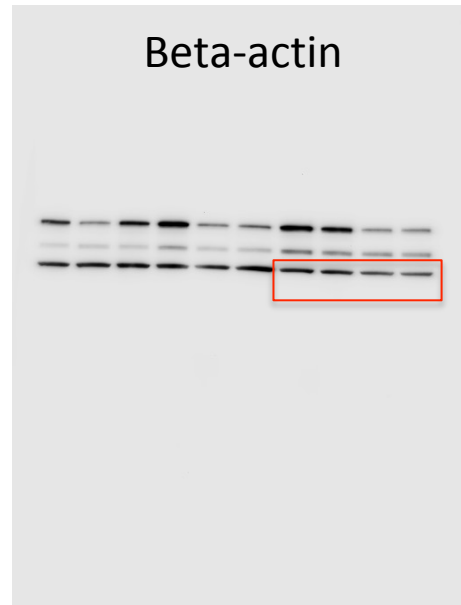

Figure.2A

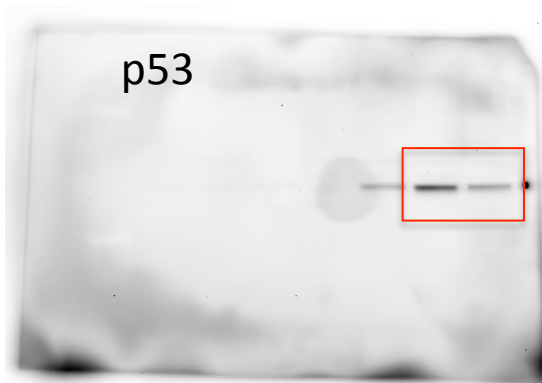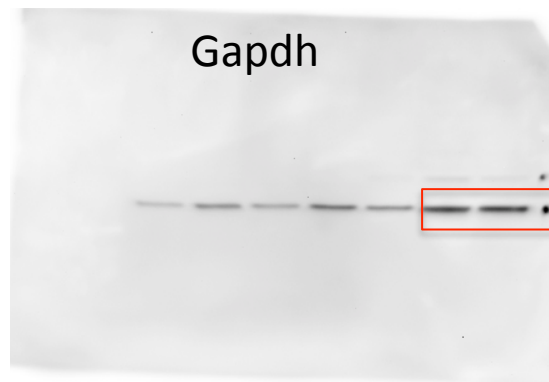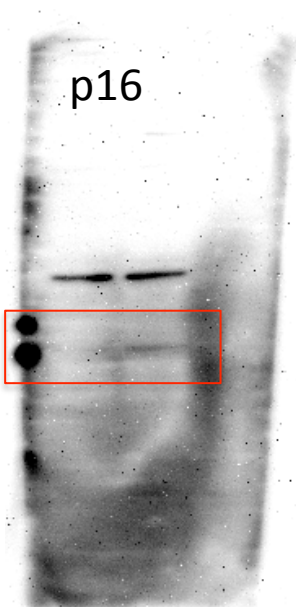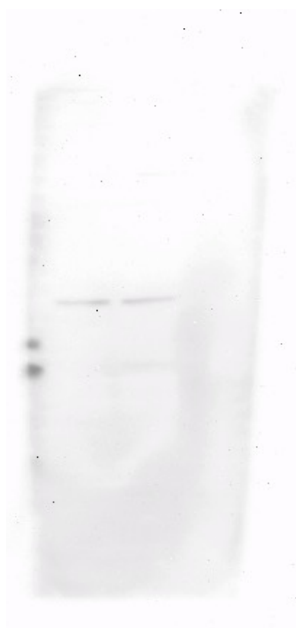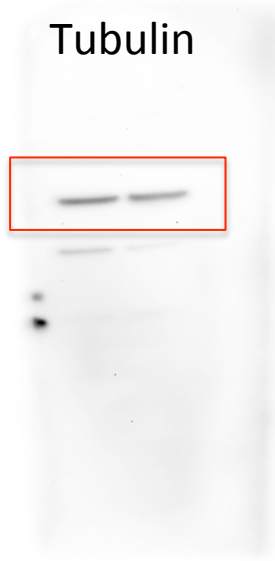

Figure.4A

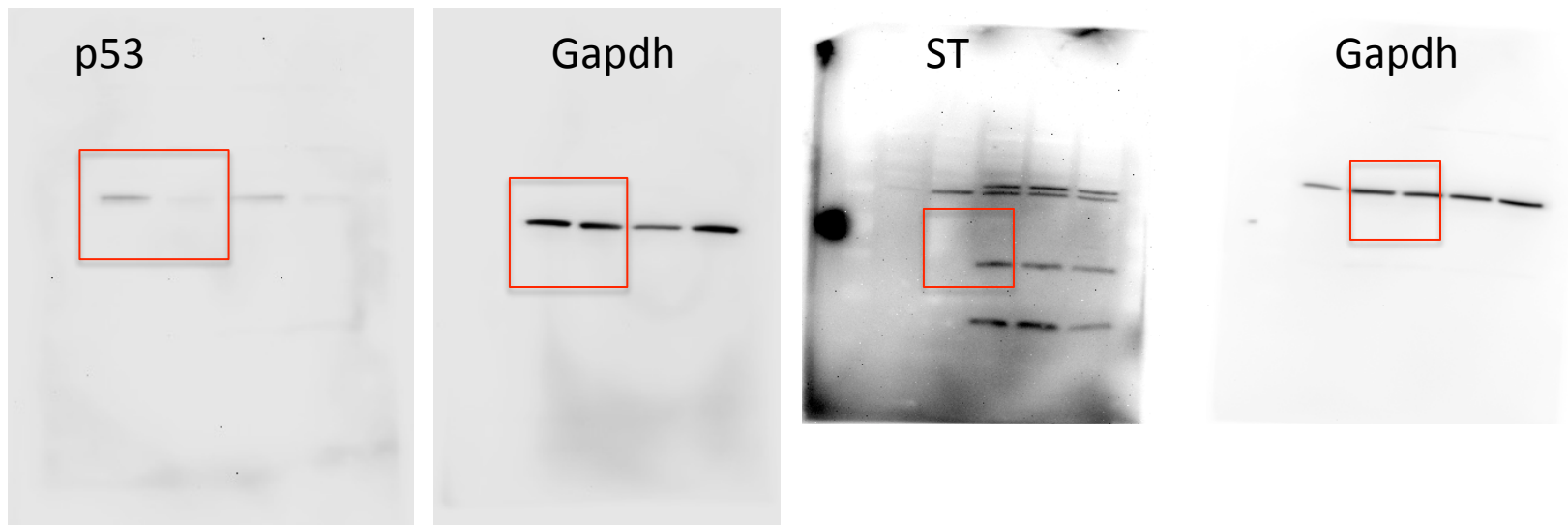

Figure.4C

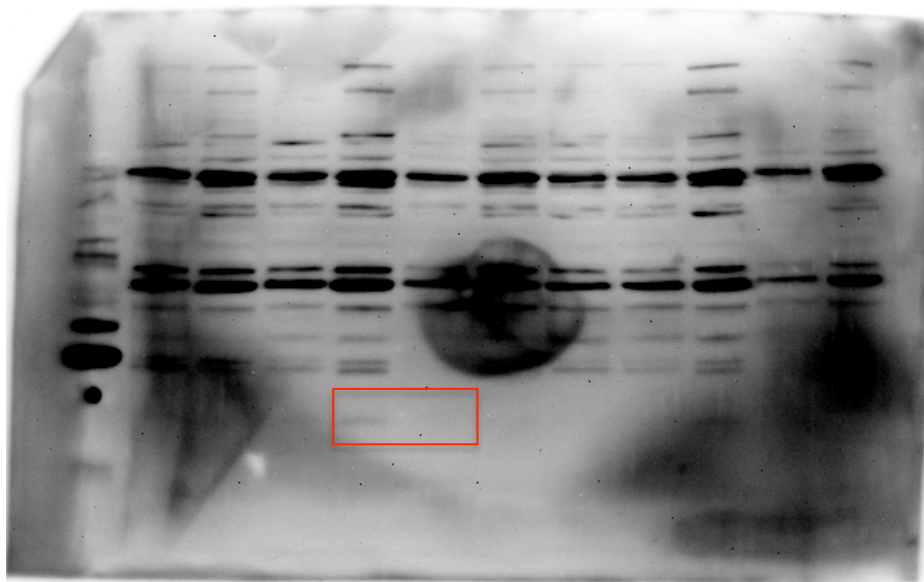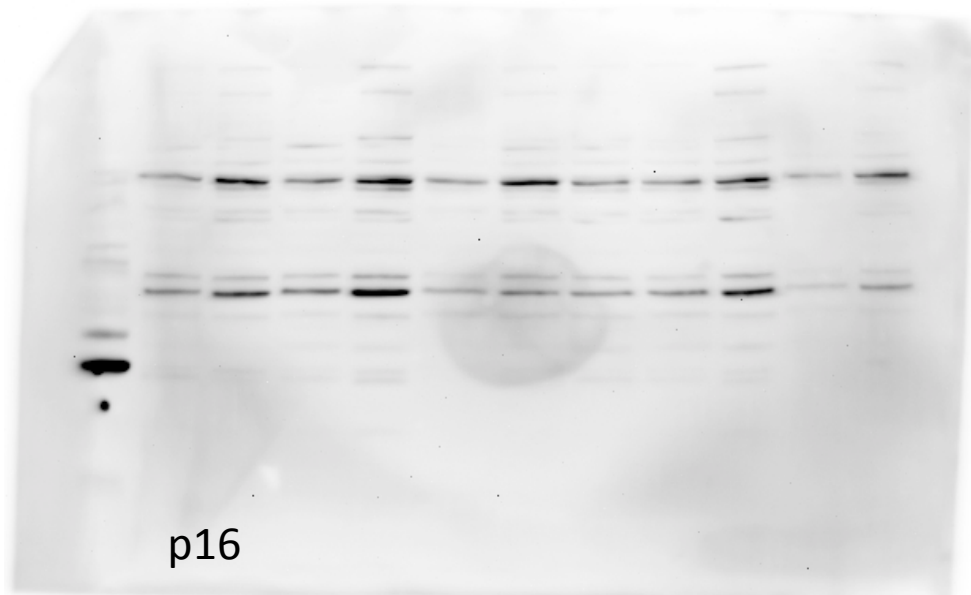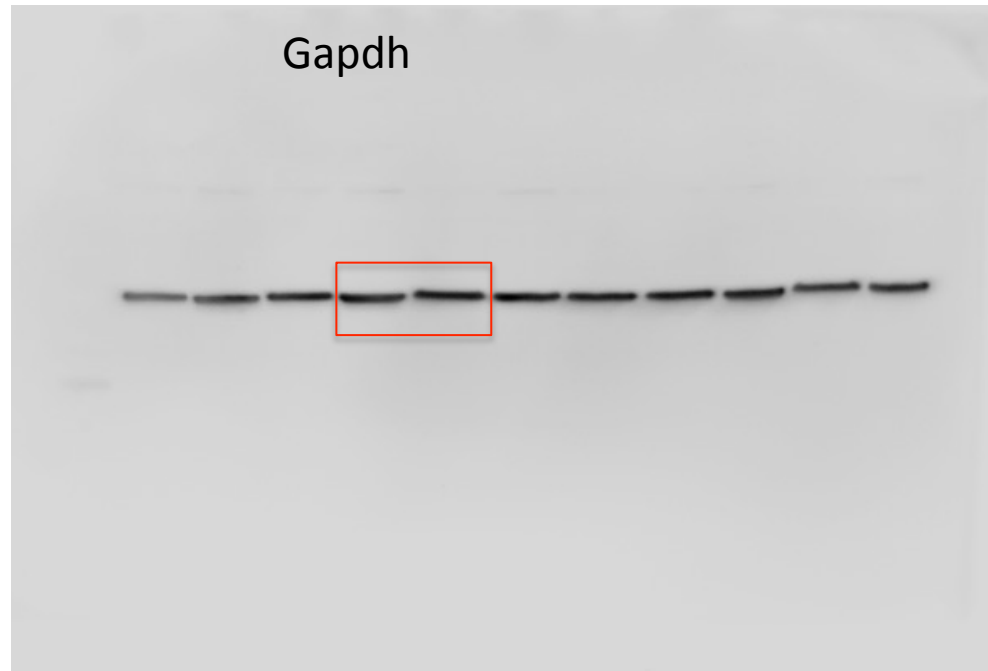

Figure.4C

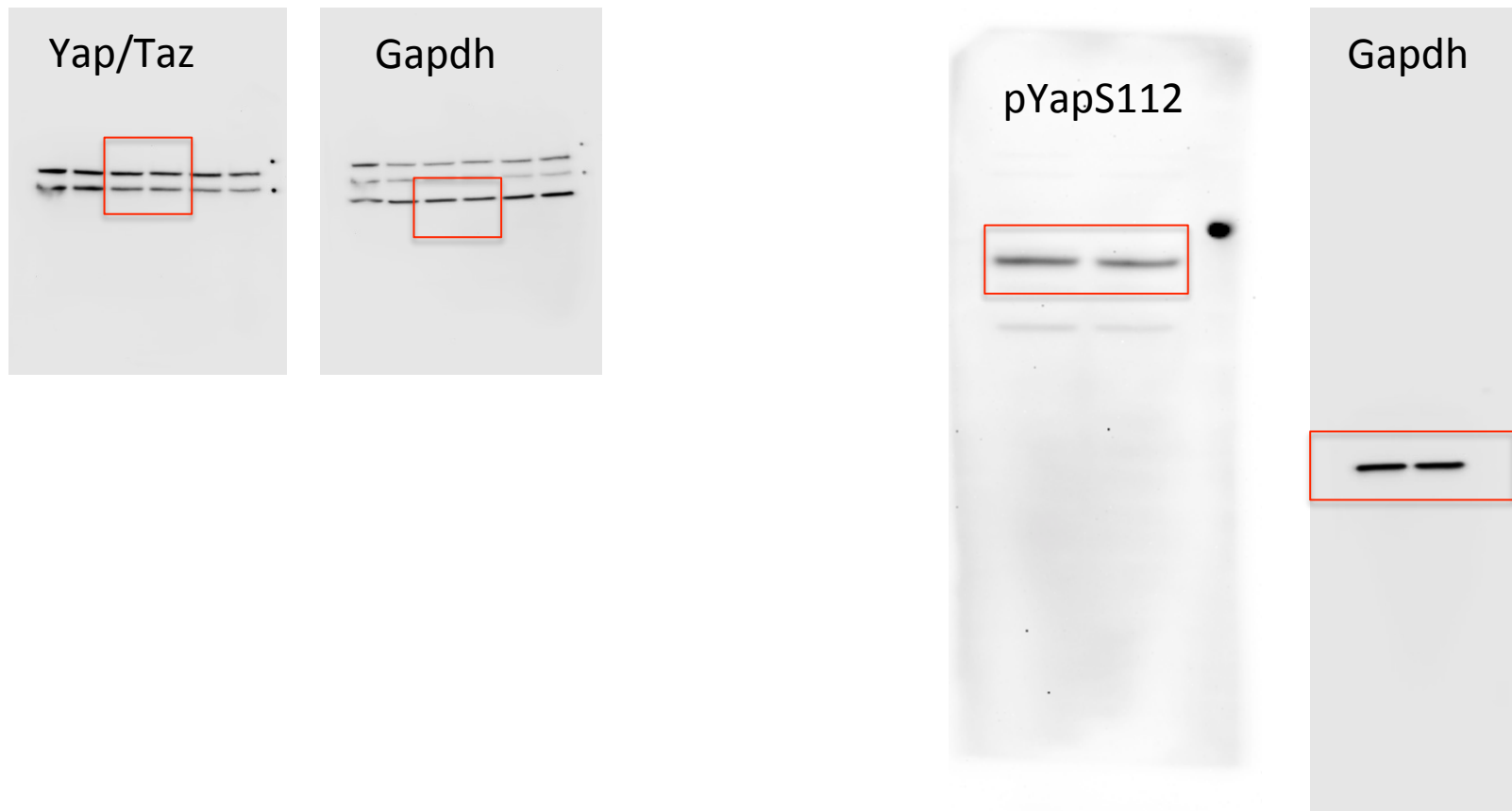

Figure.4D

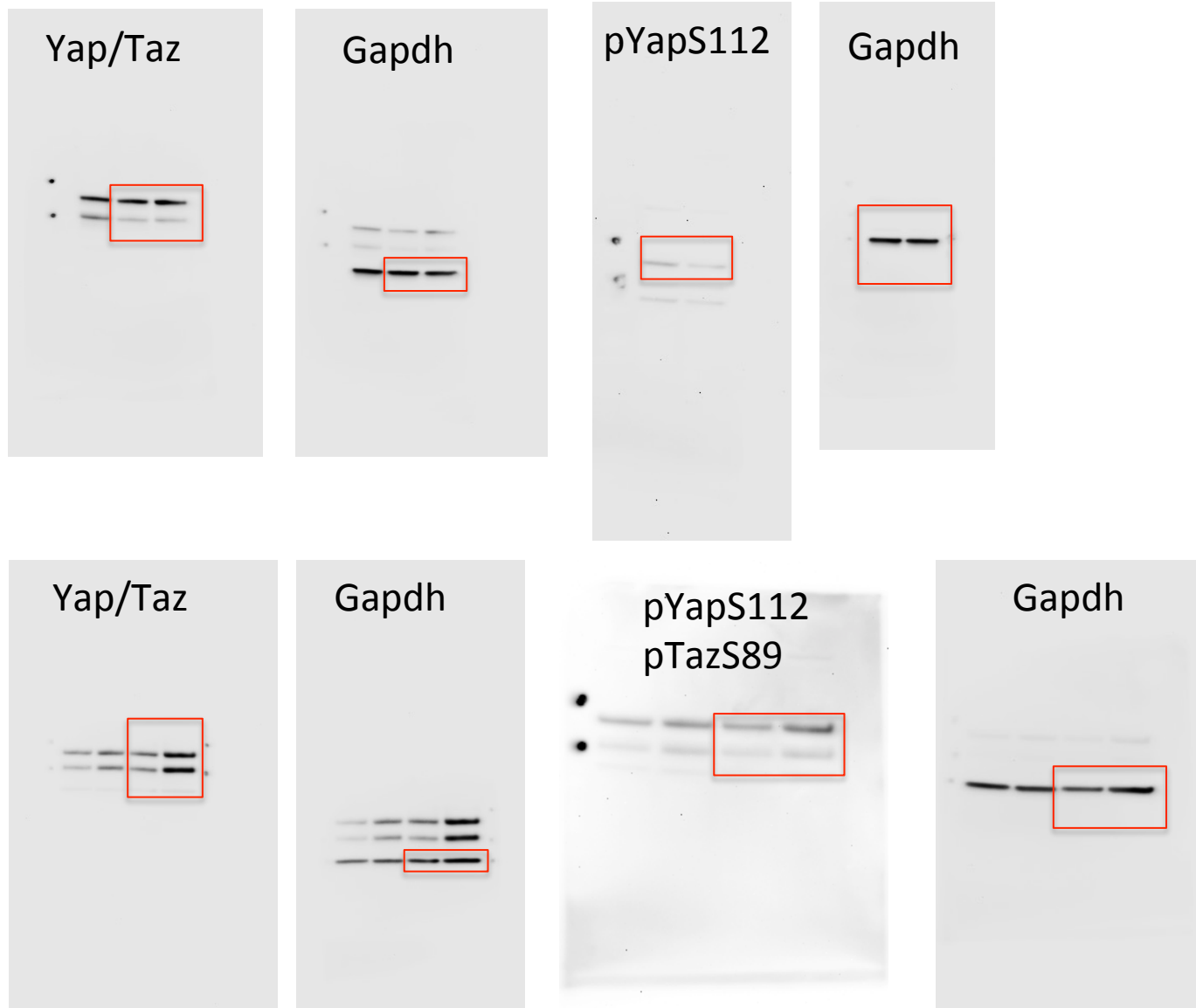

Figure.5

Supplement: Supplementary file 2 — Full western blots [file 41598_2018_33852_MOESM2_ESM.pdf]
